# Toxic Damage Increases Angiogenesis and Metastasis in Fibrotic Livers via PECAM-1

**DOI:** 10.1155/2014/712893

**Published:** 2014-03-06

**Authors:** Esther Raskopf, Maria Angeles Gonzalez Carmona, Christina Jay Van Cayzeele, Christian Strassburg, Tilman Sauerbruch, Volker Schmitz

**Affiliations:** ^1^Department of Inner Medicine, University Hospital Bonn, Sigmund-Freud-Straße 25, 53107 Bonn, Germany; ^2^Krankenhaus Marienwörth, Mühlenstraße 39, 55543 Bad Kreuznach, Germany

## Abstract

Excessive ethanol consumption is one of the main causes of liver fibrosis. However, direct effects of ethanol exposure on endothelial cells and their contribution to fibrogenesis and metastasis were not investigated. Therefore we analysed whether ethanol directly affects endothelial cells and if this plays a role during fibrogenesis and metastasis in the liver. Murine and human endothelial cells were exposed to ethanol for up to 72 hours. *In vitro*, effects on VEGF, HIF-1alpha, PECAM-1, and endothelial cell functions were analysed. *In vivo*, effects of continuous liver damage on blood vessel formation and metastasis were analysed by PECAM-1 immunohistochemistry. Ethanol increased HIF-1alpha and VEGF levels in murine and human endothelial cells. This resulted in enhanced intracellular signal transduction, and PECAM-1 expression as well as tube formation and wound healing. *In vivo*, toxic liver damage increased angiogenesis during fibrogenesis. Metastasis was also enhanced in fibrotic livers and located to PECAM-1 positive blood vessels compared to nonfibrotic mice. In conclusion, ethanol had strong effects on endothelial cells, which—at least in part—led to a profibrotic and prometastatic environment mediated by PECAM-1. Blockade of increased PECAM-1 expression could be a promising tool to inhibit fibrogenesis and metastasis in the liver.

## 1. Introduction

Fibrosis and cirrhosis, respectively, are currently the tenth most common cause of death in western countries [1]. One of the main causes of liver fibrosis/cirrhosis besides chronic hepatitis infection or hemochromatosis is excessive alcohol consumption [2]. This leads to repeated damage/wound healing of the liver. During this process, hepatic stellate cells (HSC) are activated and contribute to the altered liver parenchyma [3, 4]. Besides the effect on HSC, liver sinusoidal cells (LSEC) are also affected by fibrogenesis. However, effects on endothelial cells of larger vessels were barely investigated. In the nonfibrotic liver, larger vessels are only found as branches of the hepatic artery or portal vein, whereas during fibrogenesis, the liver structure is altered and portal areas and septae are formed, which could originate from endothelial cells activated by proangiogenic chemokines.

Angiogenesis is a well-established mechanism during fibrogenesis of the liver. Fibrosis is characterised by intrahepatic vascular remodelling, with capillarisation of sinusoids, fibrogenesis, and development of intrahepatic shunts [5, 6]. This leads to hypoxia in the inflamed liver, which results in the increased expression of the hypoxia inducible factor HIF-1alpha, which in turn transactivates VEGF (vascular endothelial growth factor) [7, 8]. VEGF is secreted and binds to its receptors on endothelial cells inducing—via intracellular signal transduction cascades—endothelial cell survival, migration, adhesion, and differentiation which leads to angiogenesis and blood vessel formation [9–12]. Recent studies suggest that pathological angiogenesis, as occurring in fibrogenesis, is a main contributor to disease progression [6, 13]. But not only fibrogenesis, but also tumour growth and metastasis are increased by fibrogenesis and angiogenesis, as was demonstrated in a previous study by our workgroup [14]. It is well known that the altered extracellular matrix promotes tumour growth and metastasis in the liver (reviewed in [15]). However, effects on endothelial cells and related cell adhesion molecules like PECAM-1 were not investigated. This is of special interest since cell adhesion molecules are known to mediate the adhesion of tumour cells to the endothelium which is a crucial step in metastasis formation [16, 17].

Therefore, we investigated if alcohol uptake directly affects endothelial cells (not liver sinusoids) and the corresponding cell adhesion molecule PECAM-1 during fibrogenesis and if this plays a role in metastasis formation. These molecular insights could contribute to the development of new antiangiogenic treatment strategies.

## 2. Materials and Methods

### 2.1. Animals and Cell Lines

Eight-week old male C3H mice were supplied by Charles River (Sulzfeld, Germany) and kept in the local central animal facility of the University Hospital Bonn. The mice were housed under standard conditions and had free access to water and food. Animal procedures were performed in accordance with approved protocols and followed recommendations for proper care and use of laboratory animals.

The murine endothelial cell line SVEC4-10 (ATCC CRL-2181) was obtained from LGC Promochem (Wesel, Germany) and cultured in DMEM supplemented with 10% FBS, 200 mM glutamine. HUVE-cells (HUVEC, pooled) were obtained from PromoCell (Heidelberg, Germany) and cultured in EGM with supplements according to the manufacturer's instructions.

Hepa129 cells (Hepatoma 129, obtained from NCI-Frederick Cancer Research and Development Centre (DCT Tumour Repository)) were maintained in RPMI1640 supplemented with 10% FBS, 200 mM glutamine.

### 2.2. Induction of Toxic Damage* In Vitro*/*In Vivo* and Tumour Cell Implantation

Murine SVEC4-10 and human HUVE cells were incubated with 100 mM ethanol for a time period of three days. Every day, a subgroup of the cells (*n* = 4 per group) was harvested and processed for further analysis.

*In vivo*, fibrosis was induced in female C3H mice according to a previous publication using TAA (200 mg/g bodyweight) and ethanol (10% in drinking water) [18]. As a control, age matched nonfibrotic mice were used. Every four weeks a subgroup of nonfibrotic and fibrotic animals were sacrificed and livers were harvested for RNA isolation and immunohistochemistry.

16 weeks after starting fibrosis induction, orthotopic tumours were induced by injecting 10^5^ Hepa129 cells in the left liver lobe as described in a previous publication [19]. After tumour cell implantation, TAA and ethanol application was prolonged.

### 2.3. Cell Viability

The cells were seeded in 96-well plates (5 × 10^3^ cells/well) and treated with ethanol for up to 72 hours (*n* = 4 per group). Untreated cells were used as controls. To analyse cell proliferation, every 24 hours a subgroup of the cells was incubated with the tetrazolium salt (MTT) and afterwards solubilized with DMSO. Optical density was determined using the GloMax Multi Reader (Promega, Mannheim) at 560 nm.

### 2.4. Analysis of Angiogenic Gene Expression* In Vitro* and* In Vivo*

SVEC4-10 or HUVE cells were seeded on 10 cm dishes. One day later, the cells were treated with 100 mM ethanol for 0, 24, 48, and 72 hours. Subsequently, protein or RNA was isolated.

Every four weeks a subgroup of fibrotic and nonfibrotic mice was sacrificed and livers were explanted for further investigation. Liver fragments (50 mm^3^) or cells were homogenised in PBS with protease inhibitors (Complete, Roche Diagnostics) or lysis buffer #11 according to previous publications [19, 20]. The whole protein content was determined and 100 *μ*g protein for the VEGF-ELISA and 200 *μ*g protein for the HIF-1alpha ELISA (both from R&D Systems, Wiesbaden, Germany) were used.

RNA from SVEC4-10 and HUVEC was isolated with the high pure RNA isolation Kit (Roche Diagnostics) and RNA from nonfibrotic and fibrotic livers was isolated with the high pure RNA tissue Kit (Roche Diagnostics). RNA was reverse transcribed (transcriptor first strand cDNA synthesis Kit, Roche Diagnostics) and a semiquantitative real time PCR for PECAM-1 (mouse left: 5′-agc cag tag cat cat ggt ca-3′, mouse right: 5′-agc agg aca ggt cca aca ac-3′, universal probe library number #25; human left: 5′-gca aca cag tcc aga tag tcg t-3′, human right: 5′-gac ctc aaa ctg ggc atc at-3′, universal probe library number #26) was done using LightCycler technique according to previous publication [21]. Relative expression levels were determined using ALAS1 as housekeeping gene. Primers were obtained from Sigma-Aldrich (Munich, Germany) and probes from Roche Diagnostics.

To analyse the localisation of PECAM-1 during fibrosis development* in vivo*, immunohistochemistry for PECAM-1 was performed. Cryopreserved liver sections from fibrotic (tumour bearing) and nonfibrotic (tumour bearing) mice were immunostained with monoclonal rat anti-mouse CD31 (eBioscience). The sections were incubated with a corresponding secondary antibody. Detection was done using streptavidin and AEC substrate (Dako cytomation, Hamburg, Germany). Sections were counterstained with hematoxylin.

### 2.5. Analysis of Intracellular Signal Transduction

The cells were incubated with ethanol as described above. Effects on intracellular signal transduction were analysed using ELISA for phosphorylated AKT, ERK, and MAPK p38 (R&D Systems, Wiesbaden, Germany). Cell lysis was performed with lysis buffer #6 according to the manufacturer's instructions. Protein content was determined with the DC protein assay according to the manufacturer's instructions. 100 *μ*g protein was subjected to the ELISA.

Effects on Stat3 were investigated in a cell based ELISA (R&D Systems). For this, 5 × 10^3^ HUVEC or SVEC4-10 were seeded on a black 96-well cell culture plate. The assay was performed in accordance with the manufacturer's protocol. Fluorescence of total and phosphorylated Stat3 was determined with the provided green and UV filter (GloMax Multi). Data was expressed as relative fluorescence units (RFU).

### 2.6. Analysis of Functional Effects on Endothelial Cells* In Vitro*

SVEC4-10 and HUVEC were incubated with ethanol as described above. 42 hours later, the cells were detached and 2.5 × 10^4^ cells were seeded on 300 *μ*L Matrigel (mixed with 100 ng/mL human or murine VEGF) in a 24-well plate. Incubation with ethanol was prolonged for another 6 hours. Tube formation was then determined using an inverted microscope by counting the whole circular tube-like structures per high power field (*n* = 12 hpf per group).

Endothelial cell migration was investigated by an* in vitro* wound healing assay. For this, 10^5^ SVEC4-10 or HUVEC were seeded in 12-well plates. 24 hours later, the cells were treated with ethanol and a scratch “wound” was applied to the cells. Ethanol damage was prolonged during the whole experiment. Every 24 hours, the cultures were analysed under a light microscope for migration of the cells in the unoccupied area. The widths of the gaps were measured at three reading points per hpf (*n* = 12 hpf/group) using Axiovision imaging software (Carl Zeiss, Jena, Germany).

### 2.7. Statistical Analysis

Data has been expressed as mean ± SEM. Diversity of different experimental groups was analysed for statistical significance by a nonparametric, two-tailed test (Mann-Whitney) for unpaired samples. Significance was calculated by the log-rank test. *P* < 0.05 has been considered to be significant.

## 3. Results

### 3.1. Cell Viability Is Slightly Increased after Treatment with Ethanol

**Figure 1:**
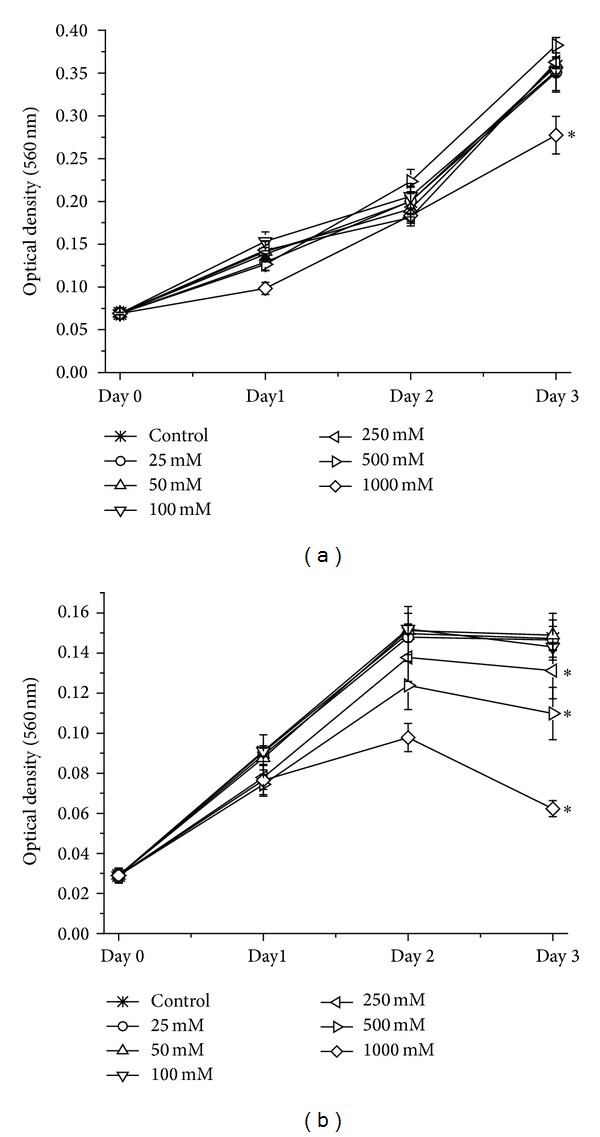
Cell viability in (a) SVEC4-10 and (b) HUVEC following toxic damage with 0−1000 mM ethanol. The cells were treated with ethanol for three consecutive days. Every 24 hours, a subgroup was stained with MTT. Optical density was determined at 560 nm. Data is expressed as mean optical density [560 nm] ± SEM. *n* = 4 per group, *P = 0.0286, compared to the corresponding control.

First, we investigated if ethanol affects overall cell viability in endothelial cells. In SVEC4-10, three days of 1000 mM ethanol damage reduced cell viability by 23% compared to untreated cells (Figure 1(a)). In HUVE cells, one day after starting ethanol treatment, 250 mM ethanol decreased cell viability by 14% compared to the control. Incubation of HUVE cells with 1000 mM ethanol for three days reduced cell viability by 58% compared to the corresponding control (Figure 1(b)). Since 100 mM ethanol was the maximum tolerable concentration, all further experiments were conducted with it.

### 3.2. Toxic Damage Increases the Expression of Proangiogenic Factors in Endothelial Cells* In Vitro*

Ethanol is known to induce hypoxia in fibrotic livers [22]. We therefore tested whether ethanol directly increased HIF-1alpha and subsequently VEGF in endothelial cells.

**Figure 2:**
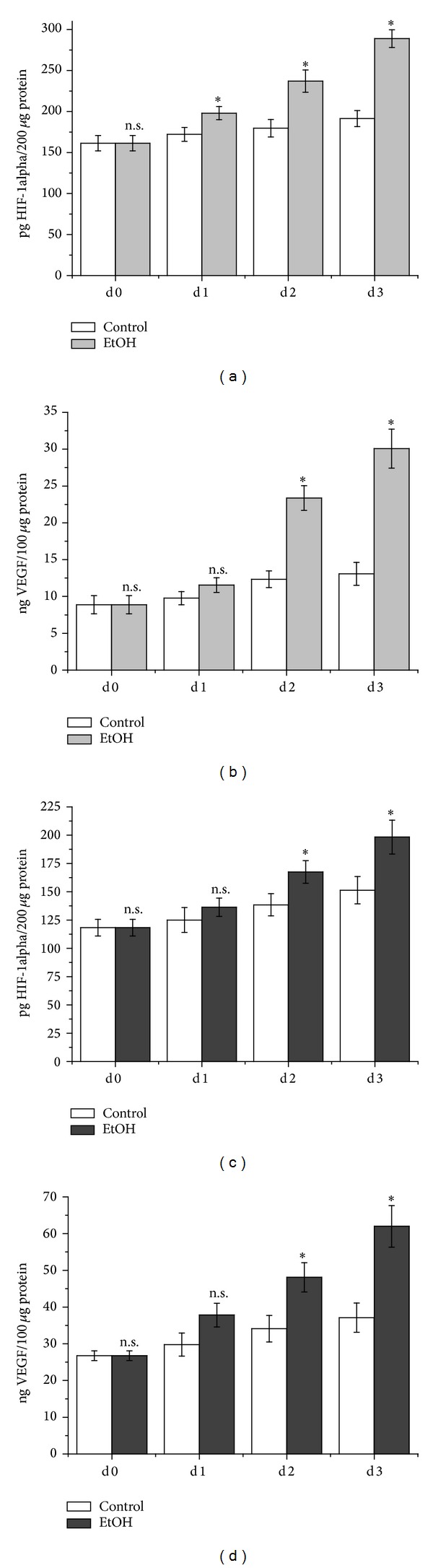
HIF-1alpha and VEGF levels in endothelial cells. SVEC4-10 ((a) and (b)) and HUVEC ((c) and (d)) following toxic damage. The cells were treated with ethanol for three consecutive days. Every 24 hours, cells were harvested and protein isolated. HIF-1a and VEGF ELISA were performed according to the manufacturer's instructions. Data is expressed as mean HIF-1alpha or VEGF content ± SEM. *n* = 4 per group, *P = 0.0286, and n.s. = not significant compared to the control.

In SVEC4-10, ethanol exposure increased HIF-1alpha by 15% compared to untreated cells. Another 24 hours later, HIF-1alpha was increased by 32% compared to the control. Three days of ethanol exposure increased HIF-1alpha by 51% compared to untreated cells (Figure 2(a)). In analogy to the increase in HIF-1alpha expression, ethanol also increased VEGF expression: one day of ethanol exposure elevated VEGF by 18% compared to the control. 48 hours of ethanol treatment increased VEGF 1.9-fold compared to untreated cells. After 72 hours of ethanol, VEGF expression was enhanced 2.3-fold compared to untreated cells (Figure 2(b)).

This was also observed in HUVE cells: 24 hours of ethanol treatment increased HIF-1alpha expression by 9% compared to the control. Another 24 hours later, HIF-1alpha was elevated by 21% compared to untreated cells. Three days of ethanol damage increased HIF-1alpha by 31% compared to undamaged cells (Figure 2(c)). This went in line with VEGF protein levels. One day following ethanol treatment, VEGF was enhanced by 27% compared to untreated cells. Two days of ethanol damage increased VEGF by 41% compared to the control. Three days of ethanol treatment elevated VEGF by 67% compared to untreated cells (Figure 2(d)).

### 3.3. Ethanol Increases the Expression of the Endothelial Cell Adhesion Molecule PECAM-1

After showing that ethanol increased proangiogenic factors like HIF-1a and VEGF, we investigated whether treatment with ethanol also affected the endothelial cell adhesion molecule PECAM-1. PECAM-1 plays a prominent role in VEGF induced endothelial cell migration and tube formation. In SVEC4-10, 24 hours after initiating ethanol treatment, PECAM RNA levels were increased 4.5-fold (0.006201 ± 0.00144) compared to the control (0.001378 ± 0.000961, *n* = 4, *P* = 0.0286). Another 24 hours later, ethanol increased PECAM expression 8-fold (0.00925 ± 0.00379) compared to the undamaged control (0.001165 ± 0.000618, *n* = 4, *P* = 0.0286). On day three of ethanol treatment, PECAM also increased 6.9-fold (0.008922 ± 0.00173) compared to untreated cells (0.001293 ± 0.000415, *n* = 4, *P* = 0.0286).

In HUVEC, similar effects were observed: 24 hours of ethanol damage increased PECAM-RNA levels 3.9-fold (0.06969 ± 0.00624) compared to untreated cells (0.01787 ± 0.00283, *n* = 4, *P* = 0.0286). Two days of ethanol exposure increased PECAM expression 6.0-fold compared to the control (0.11586 ± 0.01287 versus 0.01931 ± 0.00121, *n* = 4, *P* = 0.0286). Another 24 hours of ethanol treatment increased PECAM 5.7-fold compared to untreated cells (0.12156 ± 0.00391 versus 0.02133 ± 0.002203, *n* = 4, *P* = 0.0286).

### 3.4. Increased Angiogenic Gene Expression Results in Increased Intracellular Signal Transduction in Endothelial Cells

After detecting an ethanol induced increase in PECAM-1 gene and protein expression, it was intriguing to investigate if this leads to enhanced intracellular signal transduction. To analyse this, we performed ELISA for phosphorylated ERK1/2, P38 MAPK, and JNK.

**Figure 3:**
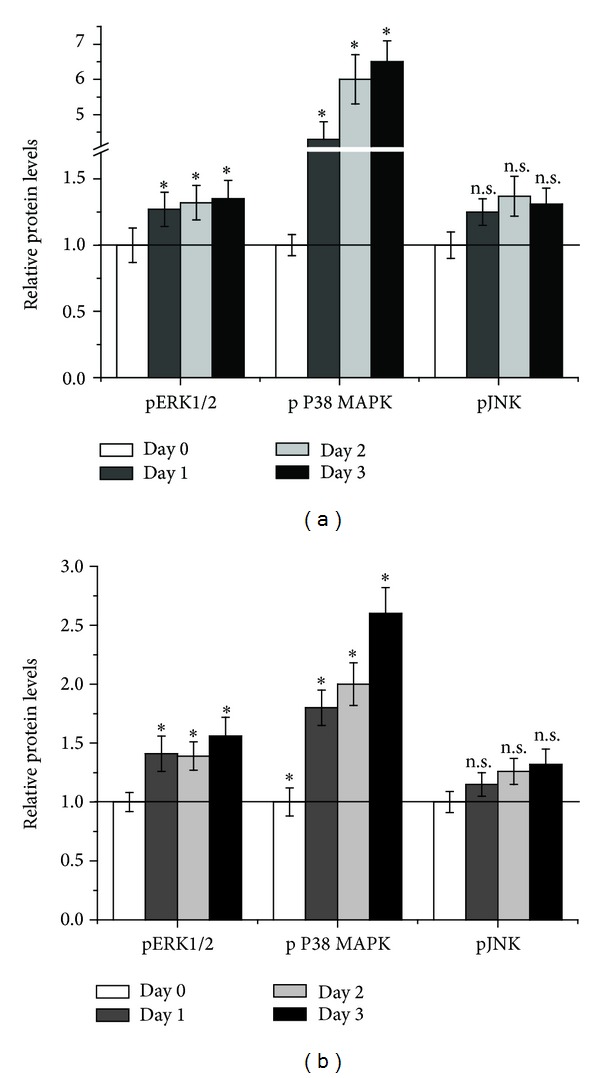
Intracellular signal transduction after ethanol damage in endothelial cells. SVEC4-10 (a) and HUVEC (b) were damaged with ethanol for three consecutive days. Every day, a subgroup of the cells was harvested and protein isolated. Protein was subjected to different ELISA, detecting phosphorylated ERK1/2, P38 MAPK or JNK. Data is expressed as relative phosphorylated protein levels ± SEM with the untreated controls set as 1. *n* = 4, *P = 0.0286, and n.s. = not significant compared to the control.

In SVEC4-10, 24 hours of ethanol increased phosphorylation of ERK1/2 by 27% compared to the controls. Another 24 hours of ethanol exposure increased pERK1/2 by 32% compared to untreated cells. 72 hours of ethanol damage further increased phosphorylation of ERK1/2 (by 35%) compared to undamaged cells. Analysis of phosphorylated P38 MAPK showed after 24 hours a 4.3-fold increase in ethanol treated cells compared to untreated controls. Two days of ethanol exposure enhanced pP38 MAPK 6-fold compared to untreated cells. 72 hours after the addition of ethanol pP38 MAPK was further increased (6.5-fold) compared to the control. Phosphorylation of JNK was also increased (by 25%) after 24 hours of ethanol exposure compared to the control. 48 hours after starting ethanol damage, pJNK was elevated by 37% compared to undamaged cells. Another 24 hours later, JNK phosphorylation was slightly reduced compared to day two but still increased (by 35%) compared to untreated control cells (Figure 3(a)).

The effects of ethanol on intracellular signal transduction were similar in HUVE cells: phosphorylation of ERK1/2 was also enhanced in HUVE cells (by 41%) after 24 hours of ethanol exposure compared to the control. Two days after initiation of ethanol damage, pERK1/2 was increased by 39% compared to undamaged cells. Another day later, pERK1/2 was elevated by 56% in ethanol treated cells compared to untreated HUVEC. Corresponding to SVEC4-10, phosphorylation of P38 MAPK also strongly increased following ethanol exposure. 24 hours of ethanol damage increased pP38 MAPK 1.8-fold compared to undamaged cells. Another 24 hours later pP38 MAPK was enhanced 2-fold in ethanol treated cells compared to the control. Three days of ethanol treatment further increased phosphorylation of P38 MAPK (2.6-fold) compared to control cells. Phosphorylation of JNK was also increased (by 15%) after 24 hours of ethanol exposure compared to the control. 48 hours after starting ethanol damage, pJNK was elevated by 26% compared to undamaged cells. Another 24 hours later, JNK phosphorylation was further enhanced (by 32%) compared to day two and control cells (Figure 3(b)).

**Figure 4:**
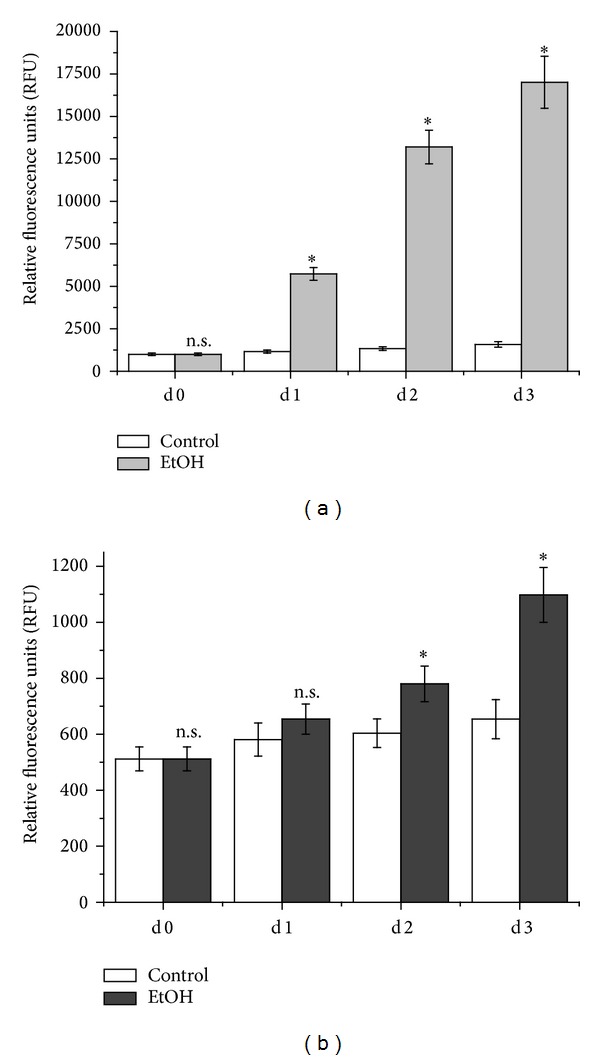
Phosphorylation of Stat3 after ethanol damage in endothelial cells. SVEC4-10 (a) and HUVEC (b) were damaged with ethanol for three consecutive days. Every day, a subgroup of the cells was subjected to the phospho-Stat3 cell based ELISA. Data is expressed as relative fluorescence units ± SEM. *n* = 4, *P = 0.0286, n.s. = not significant compared to the controls.

PECAM-1 is thought to induce Stat3 phosphorylation [23]. Here, phosphorylation of Stat3 as determined by a fluorescent cell based ELISA showed that one day of ethanol exposure increased pStat3 5-fold compared to untreated cells. Two and three days of ethanol damage increased phosphorylated Stat3 in SVEC4-10 10-fold compared to the untreated controls (Figure 4(a)). In HUVEC, one day of ethanol treatment increased pStat3 by 13% compared to the control. Two days of ethanol damage increased phosphorylation of Stat3 by 30% compared to untreated cells. Another day later ethanol enhanced pStat3 1.6-fold compared to the control (Figure 4(b)).

### 3.5. Enhanced Intracellular Signal Transduction Results in Increased Tube Formation and Migration* In Vitro*

The phosphorylation of, for example, ERK1/2 and P38 MAPK leads to increased migration and tube formation in endothelial cells. We therefore tested whether the enhanced intracellular signal transduction shown here resulted also in increased tube formation and migration.

**Figure 5:**
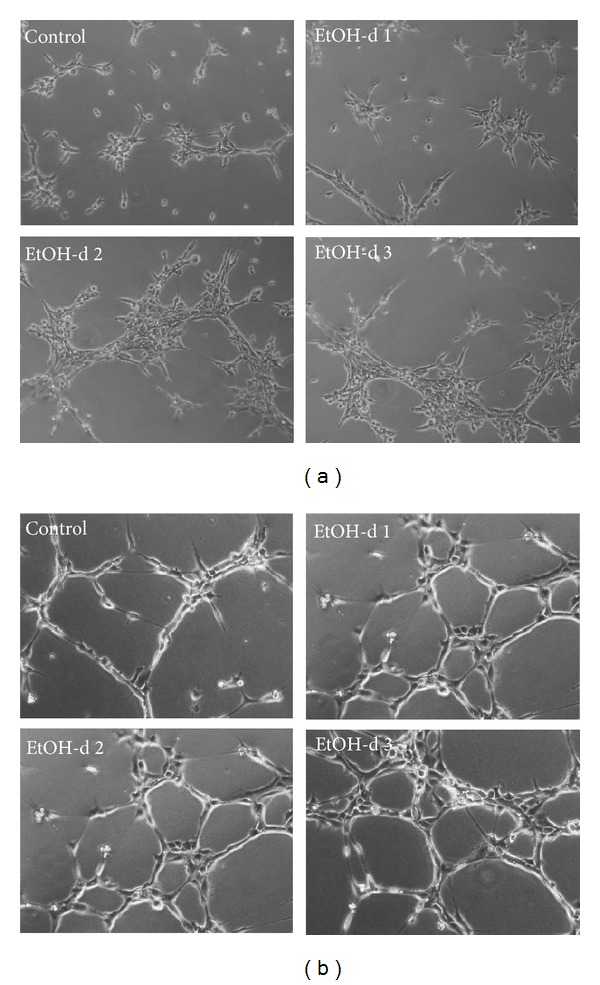
Matrigel assay of (a) SVEC4-10 and (b) HUVEC treated with ethanol. The cells were treated with ethanol for 20, 44 and 68 hours. Then, the cells were seeded on Matrigel and incubation with ethanol was prolonged for another 4 hours. Exemplary microphotographs, magnification: 100x.

After 24 hours of ethanol exposure, tube formation was increased 1.6-fold (39.5 tubes/well ± 9.7) in SVEC4-10 compared to untreated cells (24.7 tubes/well ± 6.8, *n* = 4, *P* = 0.0286). Another 24 hours later, tube formation was increased 2.7-fold (75.5 tubes/well ± 9.7) compared to the control (28.3 tubes/well ± 10.3, *n* = 4, *P* = 0.0286). Three days of ethanol exposure also increased tube formation (2.1-fold, 55.7 tubes/well ± 8.2) compared to control cells (26.5 tubes/well ± 4.9, *n* = 4, *P* = 0.0286, Figure 5(a)).

Similar results were obtained for HUVEC: 24 hours of ethanol treatment increased tube formation 1.5-fold (31.0 tubes/well ± 3.9) compared to the control (20.7 tubes/well ± 3.1, *n* = 4, *P* = 0.0286). Ethanol also increased tube formation 1.8-fold (30.3 tubes/well ± 4.9) compared to untreated cells (16.8 tubes/well ± 2.2, *n* = 12, *P* < 0.05) after 48 hours of ethanol exposure. Another 24 hours later, ethanol enhanced tube formation 1.9-fold (35.0 tubes/well ± 4.7) compared to the control (18.4 tubes/well ± 3.6, *n* = 12, *P* < 0.05, Figure 5(b)).

**Figure 6:**
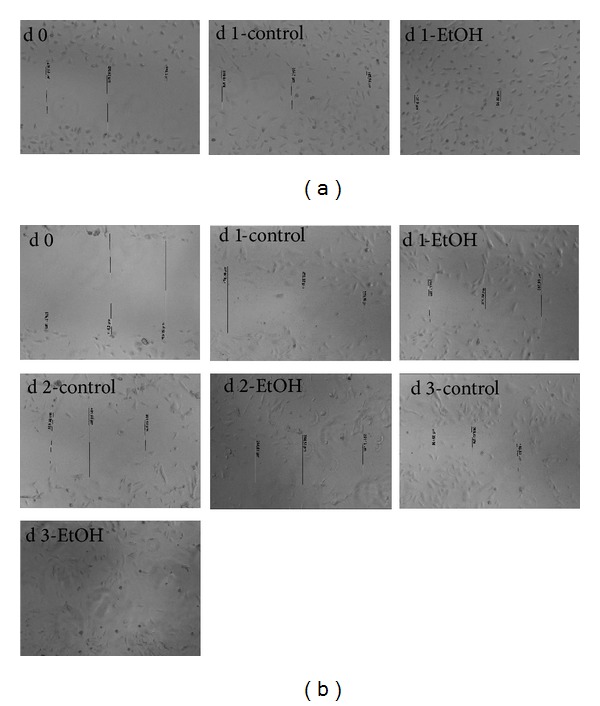
Wound healing assay of (a) SVEC4-10 and (b) HUVE cells. The cells were treated with ethanol and a scratch was made on the bottom of the culture plate. Every 24 hours, the gap was measured for migrating cells. Exemplary microphotographs, magnification: 50x.

Wound healing was also increased in both cell lines. In SVEC4-10, ethanol increased the closure of the “wound” by 89% (21.86 *μ*m ± 15.01 *μ*m) after 24 hours compared to the control (191.99 *μ*m ± 38.41 *μ*m, *n* = 12, *P* < 0.0001). After two days of ethanol damage, no measurable gap in ethanol treated and control cells was detected (Figure 6(a)). In HUVE cells, the wound closure was enhanced by 31% (333.6 *μ*m ± 9.9 *μ*m) after 24 hours of ethanol damage compared to the untreated control (483.3 *μ*m ± 24.7 *μ*m, *n* = 12, *P* < 0.05). Two days after initiating ethanol damage, the gap was further reduced (by 46%, 194.1 *μ*m ± 44.4 *μ*m) compared to the control (357.4 *μ*m ± 25.3 *μ*m, *n* = 12, *P* < 0.05). After 72 hours of ethanol damage, the gap was no longer detectable in ethanol treated cells, whereas, in the controls, a small gap (117.5 *μ*m ± 32.2 *μ*m, *n* = 12, *P* < 0.05) was still visible (Figure 6(b)).

### 3.6. Endothelial Cells Establish New Blood Vessels during Fibrogenesis

Ethanol exposure plays a prominent role in the development of fibrosis in western countries. Therefore, we tested whether development of fibrosis in a mouse model showed also altered PECAM expression. This could contribute—among other things—to a protumoural environment.

**Figure 7:**
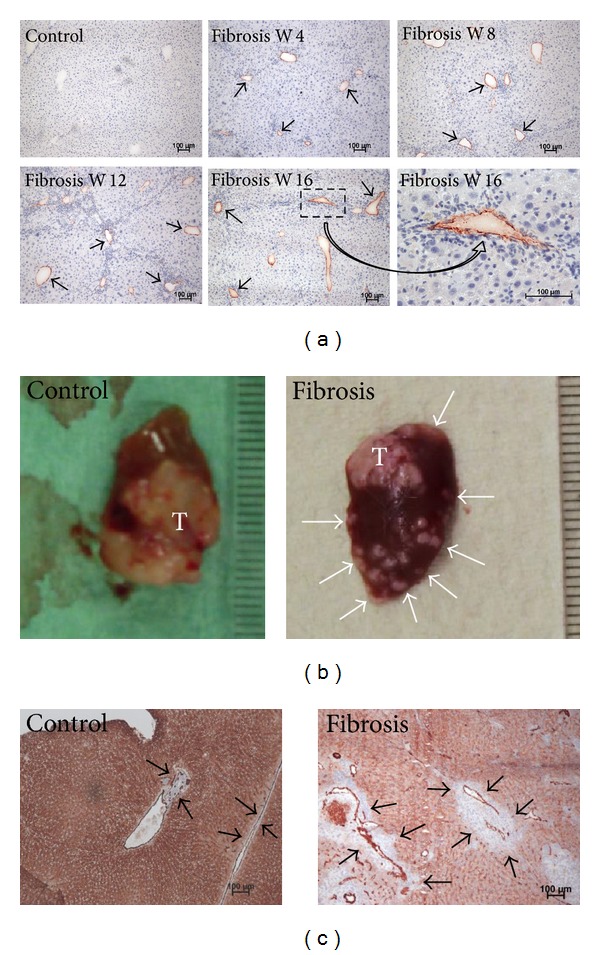
Fibrosis development and metastasis formation. Mice were treated with ethanol and TAA. Every four weeks a subgroup of the animals were sacrificed and the livers explanted. 16 weeks after starting toxic damage, Hepa129 tumour cells were implanted. Ten days later, tumour bearing livers were explanted. Liver fragments were subjected to immunohistological staining. (a) PECAM-1 expression during fibrogenesis. Positive cells show a red-brownish colour. Blood vessels are indicated by a black arrow. (b) Exemplary macroscopic photographs of hepatomas in nonfibrotic and fibrotic livers. Metastases are indicated by white arrows. (c) Exemplary microscopic photographs of tumour bearing livers. Metastases adjacent to blood vessels (red brownish colour) are indicated by black arrows. Liver tissue is also stained red because of the peroxidase activity in the liver, which was not blocked in these samples.

Development of fibrosis was confirmed by van Gieson staining (data not shown). After four weeks of toxic liver damage, PECAM-1 gene expression increased 1.8-fold compared to the control. Another eight and twelve weeks after initiation of liver damage, PECAM-1 was elevated 1.4-fold and 1.6-fold compared to the corresponding nonfibrotic control. Sixteen weeks of liver damage increased PECAM-1 2-fold compared to untreated animals (Table 1). Detection of PECAM-1 by immunohistochemistry revealed that the cell adhesion molecule was mainly located in the developing portal tracts during fibrogenesis with increasing staining intensity (Figure 7(a)).

Ten days after tumour cell implantation, fibrotic livers showed larger tumours and more metastases compared to nonfibrotic livers, which mainly possessed one singular tumour (Figure 7(b)). Microscopic analysis showed that, in fibrotic livers, metastases were mainly located adjacent to blood vessels, whereas, in nonfibrotic animals, metastasis formation was not detected (Figure 7(c)).

## 4. Discussion

Liver cirrhosis is currently the tenth most common cause of death in western countries with alcohol being one of the main inducers of fibrosis/cirrhosis [1, 2]. On the molecular level, the process of repeated damage and repair activates HSC, leading to defenestration of liver sinusoids and excessive alteration of the extracellular matrix [5, 24]. However, direct effects of ethanol on larger blood vessels and the corresponding endothelial cells were barely investigated although increased blood vessel formation increases metastatic risk [25, 26].

Therefore, we analysed whether ethanol damage directly affected endothelial cells and angiogenesis* in vitro* and* in vivo*. For this, effects of ethanol on HIF-1alpha, VEGF, intracellular signal transduction, and proangiogenic properties of murine and human endothelial cells were analysed. Angiogenesis* in vivo* was investigated in a murine fibrosis model by staining endothelial vessels for PECAM-1 (CD31). To test if increased angiogenesis also leads to increased metastasis, we applied an orthotopic tumour model.

Toxic liver damage elevated HIF-1alpha and VEGF protein levels, which not only resulted in enhanced intracellular signal transduction, but also led to increased proangiogenic effects like wound healing and tube formation.* In vivo*, staining intensity of PECAM-1 increased during fibrogenesis and was localised at the evolving portal areas. In fibrogenic animals metastasis was increased and localised adjacent to blood vessels. Further analysis showed that PECAM-1 expression was not only increased in the portal areas, but also in endothelial cells themselves after ethanol damage* in vitro*.

Ethanol was used to simulate fibrosis* in vitro*. Since ethanol is toxic, we first investigated if ethanol affected cell viability. Administration of 100 mM ethanol over a time period of three days did not considerably affect cell viability. We chose this moderate alcohol concentration to analyse “long term” effects* in vitro*. Despite this moderate alcohol concentration, HIF-alpha and particularly VEGF were strongly increased after ethanol treatment. That HIF-1a is increased following ethanol damage was shown in liver sinusoidal endothelial cells derived from ethanol fed rats and in human dermal microvascular cells [27]. Some studies propose—in analogy to, for example, tumour cells—an autocrine loop in endothelial cells due to hypoxic stress [28]. Loss of HIF-1alpha inhibits VEGF expression and endothelial cell functions [29]. So, an increase in HIF-1alpha, as shown in the presented study, could display reciprocal effects in the analysed endothelial cells.

In line with this, increased VEGF also increased intracellular signal transduction. Mainly, molecules leading to increased endothelial cell functions, like migration, adhesion, and tube formation were phosphorylated. We also analysed phosphorylation of Stat3, since pStat3 is known to activate VEGF expression [30]. Stat3 phosphorylation was increased as well, going in line with the increased VEGF levels. In another study, targeting Stat3 by using a small molecule inhibitor blocked not only VEGF, but HIF-1alpha as well [30], being another hint for autocrine mechanisms in the analysed endothelial cells. The enhanced intracellular signal transduction resulted in increased endothelial cell migration and adhesion as was determined by a wound healing assay and a matrigel assay. This data confirms results from a previous study by Morrow et al.: after 24 hours of incubation with 25 mM ethanol, tube formation and wound healing was increased in HUVE cells compared to untreated cells [31]. However, in this study effects were analysed for 24 hours.

After demonstrating that toxic damage using ethanol had proangiogenic effects* in vitro*, we transferred the results in an* in vivo* model. Fibrosis in mice was induced with intraperitoneal injections of TAA and additional ethanol feeding. Previous studies by our workgroup showed that fibrosis develops within 10 to 15 weeks [18]. In these studies, we focused on fibrotic gene and protein expression, like the intercellular adhesion molecule ICAM and matrix metalloproteases [14, 18]. Here, we focused on angiogenesis: immunohistochemistry showed increasing staining intensity of PECAM-1 in the portal tracts during fibrogenesis. PECAM-1 is mainly expressed on endothelial cells where it mediates cell-cell contacts, vascular permeability, coagulation, and transmigration of leukocytes [32]. Furthermore, it plays a major role in angiogenesis and inflammation [33, 34]. PECAM-1 was not detected in the septae or sinusoids in our study. Couvelard et al. also showed that LSEC do not express PECAM-1 independent of toxic damage [35]. Since in the control group no PECAM-1 was detected and bearing in mind that ethanol did not affect endothelial cell proliferation or apoptosis, we hypothesize that these effects were due to increased migration of endothelial cells as was shown* in vitro*. This led us to the assumption that PECAM-1 could also mediate effects in fibrogenesis. That PECAM-1 plays a role during fibrogenesis was already demonstrated [1].

Increased angiogenesis is also linked with an increased risk of metastasis [25, 26]. So, we tested whether increased angiogenesis as shown in our study here also led to increased metastasis. In analogy to a previous study by our workgroup [14], tumour growth and metastasis were increased compared to tumour bearing nonfibrotic animals. In the previous study, focus was laid on proangiogenic factors, like VEGF and its receptors, whereas effects on the cell adhesion molecule PECAM-1 as a mediator of angiogenesis and metastasis were not investigated. Here, we could show that tumour cells were mainly located adjacent to PECAM-1 positive blood vessels in fibrotic livers, further highlighting the role of PECAM-1.

The increase of PECAM-1 during fibrogenesis could be due to an either increased number of endothelial cells or increased PECAM-1 gene and protein expression on endothelial cells. Therefore, we also investigated if ethanol directly affected PECAM-1 expression on endothelial cells. Surprisingly, PECAM-1 was increased after treatment with ethanol in both cell lines. In a previous study, increased VEGF induces TNF-alpha which in turn resulted in increased PECAM-expression. Furthermore, it is postulated that Stat3 is activated by PECAM-1, providing an alternative mechanism: Ilan and coworkers showed that PECAM-1 mediates Stat family signal transduction [23]. This could contribute to the effects of ethanol observed in our study. PECAM-1 could also—at least in part—mediate endothelial cell migration and capillary morphogenesis/tube formation. This was already demonstrated for mouse kidney endothelial cells. These cells derived from PECAM−/− mice showed reduced abilities to form tubes and migrate compared to PECAM+/+ mice [36].

After showing that PECAM-1 plays a role during fibrogenesis, the therapeutic potential of PECAM-1 has now to be investigated. As was shown for VEGF, VEGF inhibition via the therapeutic antibody bevacizumab had inhibitory effects on fibrogenesis [37], as well as the application of VEGF expressing plasmid vector [38]. As the role of VEGF is controversial regarding the treatment of fibrosis, we hypothesize that it is the same with PECAM-1. During fibrogenesis, defenestration occurs with increasing resistance to blood flow [6, 39]. Therefore, blockade of PECAM-1 and decreased blood vessel formation could lead to increased portal hypertension. On the other hand, blockade of PECAM-1 could be beneficial regarding tumour growth and metastasis in the fibrotic liver. Further investigations have to clarify the therapeutic role of PECAM-1 in the treatment of fibrosis and correlating tumour growth.

## 5. Conclusion

In conclusion, ethanol activates endothelial cells resulting in increased angiogenesis. This leads to a profibrotic and prometastatic environment, which is—at least in part—mediated by increased PECAM-1 expression on endothelial cells. This in turn results in increased metastasis formation in the fibrotic liver. Blockade of PECAM-1 by, for example, RNA interference could be a promising tool to inhibit fibrogenesis and metastasis in the liver.

## Figures and Tables

**Table 1:** Relative expression levels of PECAM-1 during fibrogenesis. Data is expressed as mean RNA levels in comparison to the housekeeping gene ALAS. *n* = 4 per group.

	Control animals	Fibrotic animals
Week 4	0.004867 ± 0.000547	0.008987 ± 0.000789*
Week 8	0.006620 ± 0.000612	0.008931 ± 0.001032^n.s.^
Week 12	0.005667 ± 0.000590	0.008207 ± 0.000856*
Week 16	0.004670 ± 0.000504	0.009203 ± 0.001005*

*P = 0.0286, n.s.: not significant.
